# The effect of nursing participation in the design of a critical care information system: a case study in a Chinese hospital

**DOI:** 10.1186/s12911-017-0569-3

**Published:** 2017-12-06

**Authors:** Yanhong Qin, Ranyun Zhou, Qiong Wu, Xiaodi Huang, Xinli Chen, Weiwei Wang, Xun Wang, Hua Xu, Jing Zheng, Siyu Qian, Changqing Bai, Ping Yu

**Affiliations:** 1 Department of Pulmonary & Critical Care Medicine, 307th Hospital of PLA, Beijing, 100071 China; 2 Department of Nursing, 307th Hospital of PLA, Beijing, 100071 China; 3 Department of Biomedical Engineering, 307th Hospital of PLA, Beijing, 100071 China; 4 Centre for IT-enabled Transformation, School of Computing and Information Technology, University of Wollongong, Wollongong, NSW 2522 Australia

**Keywords:** Critical care, Intensive care, ICU, Information system, Electronic health records, EHR, Nursing documentation, Nurse, Implementation, User-centred design, Participatory design, Evaluation

## Abstract

**Background:**

Intensive care information systems (ICIS) are continuously evolving to meet the ever changing information needs of intensive care units (ICUs), providing the backbone for a safe, intelligent and efficient patient care environment. Although beneficial for the international advancement in building smart environments to transform ICU services, knowledge about the contemporary development of ICIS worldwide, their usage and impacts is limited. This study aimed to fill this knowledge gap by researching the development and implementation of an ICIS in a Chinese hospital, nurses’ use of the system, and the impact of system use on critical care nursing processes and outcomes.

**Methods:**

This descriptive case study was conducted in a 14-bed Respiratory ICU in a tertiary hospital in Beijing. Participative design was the method used for ICU nurses, hospital IT department and a software company to collaboratively research and develop the ICIS. Focus group discussions were conducted to understand the subjective perceptions of the nurses toward the ICIS. Nursing documentation time and quality were compared before and after system implementation. ICU nursing performance was extracted from the annual nursing performance data collected by the hospital.

**Results:**

A participative design process was followed by the nurses in the ICU, the hospital IT staff and the software engineers in the company to develop and implement a highly useful ICIS. Nursing documentation was fully digitized and was significantly improved in quality and efficiency. The wrong data, missing data items and calculation errors were significantly reduced. Nurses spent more time on direct patient care after the introduction of the ICIS. The accuracy and efficiency of medication administration was also improved. The outcome was improvement in ward nursing performance as measured by ward management, routine nursing practices, disinfection and isolation, infection rate and mortality rate.

**Conclusions:**

Nurses in this ICU unit in China actively participated in the ICIS development and fully used the system to document care. Introduction of the ICIS led to significant improvement in quality and efficiency in nursing documentation, medication order transcription and administration. It allowed nurses to spend more time with patients to improve quality of care. These led to improvement in overall nursing performance. Further study should investigate how the ICIS system contributes to the improvement in decision making of ICU nurses and intensivists.

## Background

With rate up to 70%, failure of health information technology (HIT) project is a widespread, significant problem [1]. The “design and reality gap” underpins HIT failure [2]. The socio-technical nature of HIT calls for its design and development to be user centred [3]. Therefore, user participation, better tools for modelling the relevant healthcare processes and better management of the development project are recommended to reduce the risks for HIT failure [3].

The first intensive care unit (ICU) in China was established in Beijing in 1982 [4]. Although intensive care medicine was only officially recognized by the Ministry of Health as a clinical specialty in 2009 [2], many ICU units have been established in hospitals around the country since then [1]. In line with the international trend of ageing population and high demand for specialised, high quality hospital services [5], intensive care is also the fastest growing hospital specialty in China.

Although only a “young” speciality in the family of hospital health care, the processes of intensive care nursing have been steadily improving, as have other specialties in Chinese hospitals. The driving force has been the government health reform program [6] underpinned by the hospital accreditation standards. Internationally, nurses in ICU face high levels of stress due to the challenges of meeting the complex needs of critically ill patients and their families [7]. Therefore, many hospitals in China are implementing strategies and innovations to optimize nursing processes to improve efficiency and patient and society satisfaction.

Translation of knowledge into rational care is as essential and pressing as the development of new diagnostic or therapeutic devices, and is arguably even more important [8]. However, a lack of information required for clinical and managerial decisions has been one of the major problems in ICUs internationally [9, 10]. As the major assembly point for critically ill patients in a hospital, each patient in the ICU will generate a large volume of data. Information and communication technology (ICT) has and will continue to play an important role in managing the data to improve patient care [8] in daily clinical services [5]. Together with engineering strategies, ICT provides an excellent opportunity to improve the design of hospital care [5].

Despite its importance, Lapinsky et al. found the use of fully integrated intensive care information systems (ICIS) was still not common in 2008 [11]; only 46% of ICUs in 50 hospitals in Canada introduced medication administration records, even fewer (26%) used computerised nursing notes.

Several authors have suggested that computerised nursing information systems are among the optimal choices to improve nursing care in ICUs [5, 12]. Bosman et al. found that the use of an ICIS in patients after cardiothoracic surgery alters nursing activity; it reduces time for documentation and increases time for patient care [13]. Hoekstra et al. found that computerized potassium control, integrated with the nurse-centred GRIP program for glucose regulation, is effective and reduces the prevalence of hypo- and hyperkalaemia in the ICU compared with physician driven potassium regulation [14]. Nyholm et al. suggested that the use of nurse checklists in a bedside computer-based information system may elevate nursing alertness to avoid secondary insults and help in the evaluation of patients’ conditions [15]. Conroy et al. found evidence of improvements in the delivery of essential daily care processes after introduction of a valid e-checklist to the morning ward rounds in an ICU [16]. Yusof finds that most nursing staff have a positive perception of an ICIS in reducing documentation and data access time, and giving them more time with patients [17].

To date there is no refereed paper reporting ICIS development, implementation, usage and impact from Asian countries including China. The international research community have no knowledge about whether and how ICIS is introduced and used by nurses in China, and what are the impacts of Chinese nurses’ use of ICIS on their work practices and performance. Lack of this knowledge is a serious impediment for the international advancement of intensive care as a medical specialty and ICIS as an effective tool to support intensive care services.

Critical care research in China is still in its infancy [4]. Although many ICU units in China have been or are in the process of computerising their clinical care processes to improve quality, safety and productivity, these clinical ICT innovations need to be rigorously evaluated to ensure they deliver the intended benefits and avoid the potential hazards and unintended negative consequences reported elsewhere [18, 19]. Therefore, taking a case study approach, this research investigates the active participation of nurses in the design, development and implementation of an ICIS in a Chinese hospital, and the effect of the ICIS introduction on nursing care processes and outcomes. In order to provide a clear conceptual model of the nurses’ participation in the ICIS design, we first review the literature on participatory design.

### Participatory design

The goal of participatory design is to establish a more human, creative and effective relationship between the actors involved in technology’s design and its use so as to generate a user-friendly digital ecosystem [20]. The participation of the intended users, including managers, internal design professionals, and users, in technology design is seen as one of the preconditions for good design in an organisational context [21]. The three key participatory design issues [22] are (1) the politics of design, (2) the nature of participation, and (3) methods, tools and techniques for carrying out such projects. The four major steps of participatory design projects are (1) initial exploration of work, (2) discovery processes, (3) prototyping [23], and (4) organizational implementation [3].

#### The politics of design

The organizational contexts are important for the success of participatory design projects [22]. These include the distribution of power in the workplace, the rationales for participation and the ways that differently positioned actors within an organization influence technology design and implementation [24].

#### The nature of participation

The nature of participation can be conceptualised in three dimensions: (1) access to relevant information [25], (2) the opportunity for taking an independent position on the problems, and (3) participation in decision making [3]. Design professionals need knowledge of the actual use context and workers need knowledge of possible technological options. These types of knowledge are developed most effectively through active cooperation between workers and designers within specific design projects. Although the projects are likely to be initiated by the managers or design professionals, a real commitment by the actors at various levels of an organisation over the direction and outcome is required for project success [21]. This calls for careful consideration of the choice of user participants and the forms of participation. The choice can be made in consideration of the motivations for participation, the scope of participation, and the resources allocated for the project. In addition, the relations between those actively take part in the project and those who do not should be carefully considered and attended to throughout the project so as to sustain the influence of the project beyond its immediate lifecycle [26].

#### Methods, tools and techniques for participatory design

Many participatory design methods have been developed, such as cooperative experimental systems development method, prototyping experiments, and work-oriented design. Cooperative experimental systems development method is characterized by a focus on active user involvement throughout the entire development process [11]. Prototyping experiments closely couple with work situations and use scenarios to transform results from early cooperative analysis/design to targeted object-oriented design, specification and realization; and design for tailorability. A “work-oriented design” approach advocates a combination of field studies of work with case-based prototyping [27].

Tools and techniques are needed to facilitate designers to learn the everyday work practices of potential users, for workers to learn about the technical options, and for both to connect current and future work practices with envisioned new technologies. The useful tools and techniques for work analysis include visualization, reviewing written materials, questionnaires, interviews and ethnographic techniques [3]. Visualizations are useful for representing the relations between technology and work [28], the same as questionnaires and interviews [3]. Reviewing written materials and observing current technologies in use can inspire the design of potential technical solutions. Ethnographic techniques such as open ended interviews and observations can generate insights into workers’ tacit knowledge and develop shared views on the work.

The tools and techniques for system design include scenarios, mock-ups, simulations of the relation between work and technology, future workshops, design games, case-based prototyping and cooperative prototyping [29, 30]. These tools and techniques can avoid the overly abstract representations of traditional design approaches and allow workers and designers to more easily experiment with various design possibilities in cost effective ways. Gronback et al. [30] advocate the use of cooperative prototyping where users and designers collectively explore the functionality and form of applications as well as their relations to the work in question. This type of cooperation requires access to adequate prototyping tools, and the availability of workers’ actual work materials for case-based prototyping. The prototypes act as “cytalysts” and “triggers” in discussion about the relations between work and technology, leading to mutual learning.

## Methods

### Case study site

The case study site is the 14-bed respiratory ICU in the Department of Pulmonary & Critical Care Medicine in 307th Hospital of PLA in Beijing, China. The hospital is a tertiary hospital with 1500 beds. The Respiratory ICU was established in 2009, the same year intensive care was recognised as a medical specialty by the Chinese Ministry of Health. In total, there were 27 nurses in the specialty. The demographic profiles of the nurses are listed in Table 1. The nurses worked in two 12 h shifts, day shift and night shift.

**Table 1:** Demographic information of the registered nurses in the ICU in May 2016

Age (Years)	No. of persons (%)
20–25	7 (26%)
26–30	17 (63%)
30–40	3 (11%)
Sex	
Female	23
Male	4
Nursing education level	
3-Year higher education diploma	14 (52%)
4-Year Bachelor	13 (48%)
Nursing work experience	
< =3 years	6 (22%)
3–5 years	15 (56%)
> 5 years	6 (22%)

### The development of the ICIS

#### Project initiation

The first author, who was the Director of Nursing (DON) in the ICU, expressed her wish of introducing certain information system to reduce the burden of nursing documentation, and to “return nurses to patients” with the Chief Information Officer (CIO) in the hospital in 2012. After initial market research including site visits to two hospitals in Beijing with sophisticated IT systems and discussions with various HIT vendors, YQ, CB and the CIO came to the conclusion that there was no readily available ICIS that could meet the needs of the ICU nursing. The team had to develop an ICIS onsite.

#### The project team

The project team was coordinated by then CIO in the hospital. YQ was responsible to provide domain knowledge and resources, five nurses (22%) in the unit (XC, WW, XW, HX and JZ) participated in the project team. A manager in the company led the technical team. The team members included one to two software engineers in the Department of Biomedical Engineering in the hospital, all of the authors for the paper except SQ and PY, and three to eight software engineers from the company at the various software development stages.

#### The politics of design

As the initiator of the ICIS development, the nursing team was actively driving the development, providing the engineers with access to the ICU knowledge and resources. An engineer could conduct ethnographic observation in the ICU upon informing the DON and without disturbance to the unit operation. Excited by the opportunity of leading the development of ICIS innovation in China, the CIO was highly enthusiastic, allocated one to two biomedical engineers in the Department of Biomedical Engineering in the hospital to the project, as well as the office space for use by the engineers in the company for software development. Motivated by the opportunity to enter the market of ICIS, the management in the company made the strategic decision to invest resources in building the ICIS, using the hospital ICU as an experiment and test site. With the appropriate administrative position in the organization, the CIO acted as the general manager for the project.

Therefore, a shared goal that united the three collaborating partners together was producing a high quality ICIS to strengthen the position of nurses in their efforts to improve working conditions and quality of work life. The initial nursing requirement for the system was automating the nursing documentation system, real-time data capture from numerous ICT equipment, and automatic generation of the one-page 24-h special nursing care form.

#### The nature of participation

The common goal motivated the team to communicate, negotiate, compromise and collaborate in the participative design process. To solve the challenge of ambiguity of requirements and difficulty in understanding the view point of each other, the engineers and the nurses really collaborated to understand each other. Patience, collegiality, and trust led to high morale, mutual learning and development.

The nursing team provided the requirements for the system, including the nursing documentation forms and data, the information flow and its accordance with nursing work processes and exchange mechanism with other hospital information systems in the other departments.

With better knowledge of the work practices of nurses, experience of automating hospital work and cost associated with the automation, the biomedical engineers in the hospital drove the requirements capture and analysis, and the translation of nursing needs to the language of the software developers in the company.

The team regularly had participatory design meetings at the Department of Biomedical Engineering in the hospital in the late afternoon for one to three hours. Any idea, issue or suggestion was discussed, the information related to the project was transparently shared in these meetings. Each individual team member took an independent position on the problems. After discussion, the final decision was a rational choice based on the analysis of various scenarios, weighing pros and cons of each scenario.

#### The four stages of the project

##### Stage 1. Initial exploration of work

This stage was quickly gone through in one-month time. The CIO and two biomedical engineers in the hospital regularly visited the ICU unit, having meetings with the DON and the nurses to understand the nurses’ work activities, purposes, procedures, routines, technologies used, and information exchange with other departments using other hospital information systems.

##### Stage 2. Discovery processes

The biomedical engineers in the hospital used the ethnographic techniques such as observing nursing work, reviewing written documents, and communicating with nurses to understand the everyday work activities and actions of nurses and to gain insights into nurses’ tacit knowledge.

They learned nursing care processes and actions; first what nurses did, then divided the nursing actions into modules and understood each action in a module. They recorded the action, its rationale, and relationship with other actions or parameters; e.g., the relationship between medication orders and patient’s health conditions. Based on the learning, they produced the requirements documentation.

The whole team worked together, either in the ICU or in the Biomedical Engineering Department, to understand and prioritize work organisation and to agree on the technical solutions. The team would discuss the documented requirements. To balance the speed of development and the quality of software, the requirements were prioritised according to the sequence of information flow and usage rate, i.e., how many users would use a function. The functions with high usage rate were given higher priority for automation.

Tools such as diagrams, drawings and nursing scenarios were often used to visualize and analyse the model of patient journey and information flow, the relationship between technology and work in these meetings and to develop shared views on nursing work and how the ICIS could be best designed to support the nursing work.

The participating nurses learned about the technical options through direct discussions with engineers in the participatory design meetings, trialling on the prototype system and referencing with their experience of using other common software applications such as Microsoft Word and online activities.

##### Stage 3. Prototyping

The system development methods included cooperative experimental systems development method, prototyping experiments and work-oriented design.

The first version of the prototype was quickly produced in two-month time, in February 2013. As the product was a direct translation of paper records to electronic records, the nurses did not see it as what they expected, i.e., an automatic system that would help nurses to manage information. It was completely abandoned because nurses did not see it as usable. This experience taught the engineering team that automation was not a simple replication of manual process, but the best possible automation of information flow and output.

An incremental, modular development was conducted to ensure quality control for each step of patient journey. A module was centred on satisfying the information needs for each step of patient journey, such as patient admission, allocation of bed and medication order. Only after a module was built, and has satisfactorily passed unit test and user test, would the development progress to the next module.

##### Stage 4. Implementation

Between February and September 2013, the ICIS was incrementally used to record the information for one patient by a nurse in the project team. The nurse recorded the patient information both on paper and in the ICIS and checked the conformance between the two types of records. The trial rolled from one patient to the second and third. Only after all the deficits were ironed out and the software functionality and workflow met the needs of ICU nursing, was the system fully implemented in the unit in September 2013. The system was fully integrated into practice by December 2013.

The onsite engineer provided training to all nurses on usage of the system. This full-time, onsite support was continued until July 2014. This had enabled any usage problems to be resolved promptly on site, any technical glitches to be ironed out quickly and the functionality of the system to be continuously improved. The nurses involved in piloting the system also acted as trainers and champions to provide peer support to the other nurses.

There was strict procedure to follow on functional change and documentation. System was only upgraded after thorough testing to ensure error free. Installation of the upgraded system was usually conducted at mid-night on week days, avoiding the peak usage period of weekends and Mondays.

### Measurement of the effects of the ICIS introduction on nursing services in the ICU

Pre- and post-implementation comparison was conducted. The outcome data collected were documentation time versus direct patient care time, auditing of paper vs electronic nursing records, and annual hospital nursing performance data.

#### Documentation time before and after implementation of the ICIS

Pre-implementation data collection was conducted between May and September 2013, before the ICIS was implemented in the ICU. The documentation time was acquired through self-reporting by nurses in the day shift, from 8 am to 8 pm. In consideration of the frequent occurrence of emergencies, an intrinsic feature of ICUs, a nurse was given five months to record their own documentation time and direct patient care time per shift, then reported the average time over five months to the Director of Nursing (DON) in the unit.

The post-implementation data was collected during March to August 2014, five months after implementation of the ICIS, when most nurses were sufficiently experienced to use the ICIS confidently.

#### Nursing records auditing

The nursing records were audited by the Director of Nursing (DON) as part of the routine nursing documentation quality control process in the hospital. Ten to fourteen nursing records were audited randomly every day to identify missing data, missing data items, calculation errors and legibility. Missing data items were identified by a comparison of a patient’s records in a day with that of previous days.

Before the ICIS introduction, YQ audited 500 paper-based nursing records in July to August 2013. After the ICIS was fully implemented, she audited 450 electronic nursing records in May to June 2014 again, as part of the routine procedure of quality assurance.

#### Annual nursing performance data collection

Nursing performance in each ward was formally evaluated by a quality assurance committee on annual basis as an important nursing quality assurance mechanism in the hospital. Six areas were assessed: ward management, routine practice, disinfection and isolation, nursing documentation, infection rate and mortality rate. Each area was measured in several domains. Each domain was, again, measured by several tangible items, each was assigned a score. The total score of all items in all domains in an area was 100. For example, the area of disinfection and isolation was measured by four domains: hand hygiene, sterilised goods management, clinical item usage and management, and ventilator usage and management. The domain of hand hygiene was again measured by five items: (1) A clear sign to notify the procedure of hand hygiene and completeness of supplies for hand wash at the sink, 1 mark; (2) Antiseptic agent to be placed close to the entrance of the monitoring ward, 1 mark; (3) Nurses were fluent with the six-step hand wash procedure, 1 mark; (4) Washing hands promptly, washing or antiseptic disinfection before and after contact with a patient, 3 marks; and (5) Correct usage of various gloves, 1 mark. Likewise, other domains were also measured by the specific, measurable items. With a standardised, reasonably rigorous method of data collection, the annual nursing performance data provided a useful indicator of changes in nursing care quality that might be related to the introduction of the ICIS.

### Ethics approval

The study was approved by the Institutional Review Board of Affiliated Hospital of Academy of Military Medical Sciences.

## Results

### Overview of the ICIS functionality

**Fig. 1:**
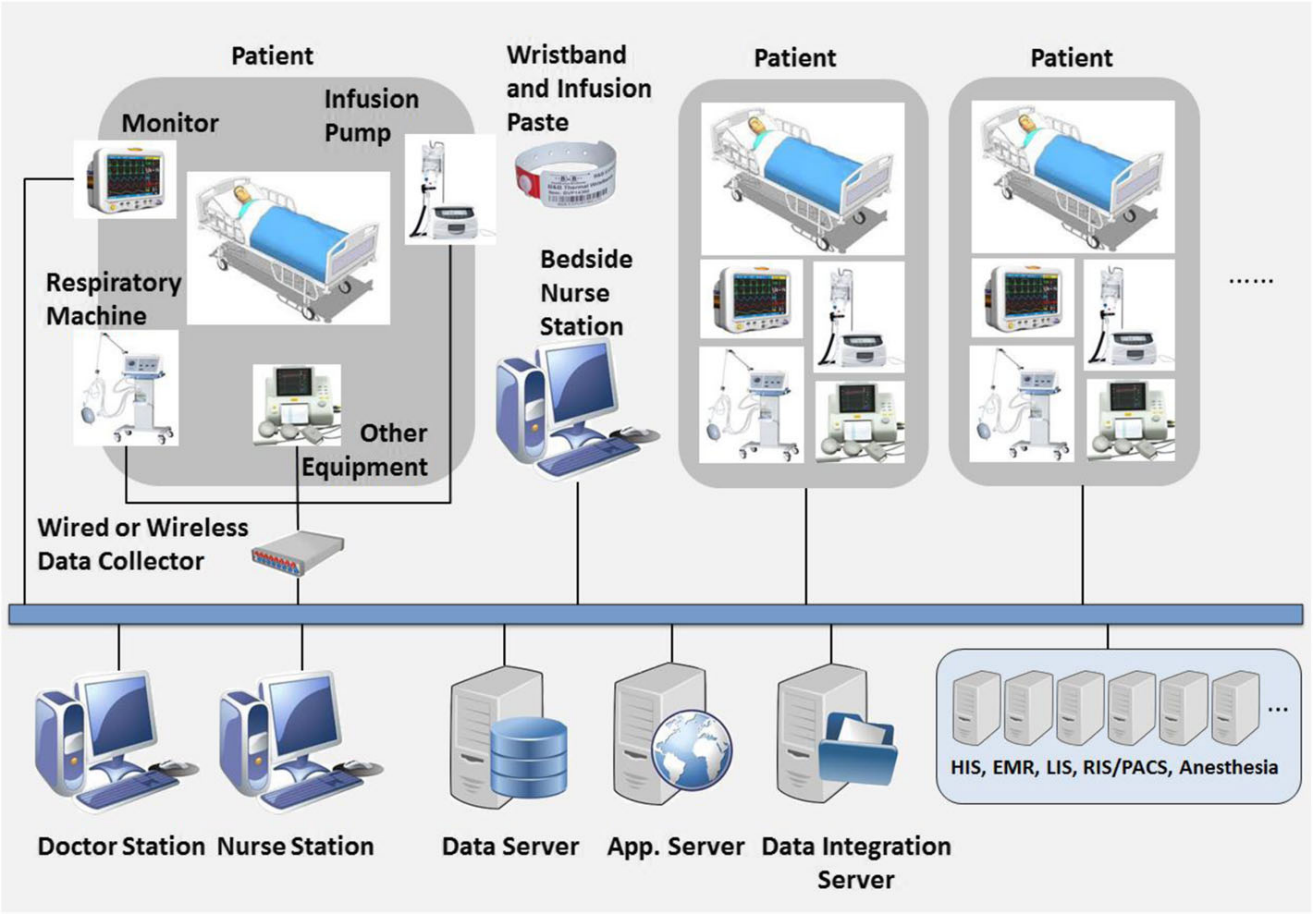
System Outline

The system had the following functionality: (1) Seamless integration with the hospital information system (HIS), picture archiving and communication system (PACS), laboratory information system (LIS) and electronic medical records (EMR); (2) Automatic collection and recording of data from bed-side monitoring devices and respirators via a centralised transmitter; (3) Extraction of physician order entries from the HIS; (4) Bar-code verification of a patient’s identity and the medication to be administered for preventing medication errors; (5) Generation of line charts of patient vital signs; (6) Automatic generation of a daily one-page, 24-h “Critical Care Chart”; (7) Provision of an interface for nurses to enter patient intake and discharge records, body temperature, nursing records and medication management records. Figure 1 presents an outline of the system.

**Fig. 2:**
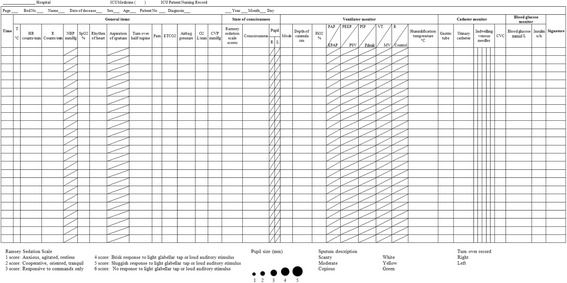
ICU Critical Nursing Care Chart

The data captured from the monitoring devices included blood pressure, heart rate, breathing, transcutaneous oxygen saturation, ventilator output, blood gas analysis parameters, and various intake and output statistics. The system automatically conducted blood gas analysis, calculated oxygenation index, hemofiltration and fluid intake and output balance. The interval of data collection is one minute. The numerical data were presented in a one-page, easy-to-read intuitive diagram called a Critical Care Chart (see Fig. 2).

There were 20 types of quantitative evaluation of clinical effects built into the ICIS. The critical disease assessment model in the ICIS included acute care physiology and chronic disease evaluation (APACHE II score), sequential organ exhaustion evaluation system (SOFA score), and comprehensive assessment of multiple organ dysfunction (MODS score).

Abnormal data captured from various monitoring devices, such as blood pressure outside normal range, or out of range data entry, were automatically identified and alerted to nurses by the ICIS. For example, the abnormal data collected from an electrocardiography would be colour-coded to draw the attention of nurses to the need for further assessment. Nurses were alerted to the depth of entrance of endotracheal intubation, if longer than the total length of the tube by a pop-up window for further validation.

It is common that 10 to 20 medications are prescribed to a patient in the ICU. In the ICIS, in order to standardise the process of medication administration, each medication in a physician order entry was automatically classified as a long-term or temporary medication by the period of medication. Long-term medications were further classified by the method of administration into three types: (1) oral medication, (2) minor treatment, such as oxygen and cardiopulmonary suctioning, and (3) injection, including intravenous and intramuscular injection.

In accordance with the characteristics of nursing in the ICU, specific forms and charts were designed in the ICIS to present real-time changes in vital signs. These included an observational chart of vital signs, critical patient records, critical nursing charts, various assessment forms and medication administration forms. The database was established to collect, integrate, analyse and export data to the end users, nurses. The system also provided search functions for all the data in the database. The data searched can be stored in an Excel spreadsheet.

### Nurses’ usage of the ICIS

The system was used in three major functional areas of nursing: data capture and entry, information output and medication administration. Nurses used mobile terminals on cart and desktop computers to access data electronically. After introduction of the ICIS, paper was no longer used by nurses in the ICU. The nurses who participated in the focus group discussions all expressed satisfaction with the ICIS and the way it was implemented, grateful for the support received from the onsite engineer. One nurse reported “The system was used to full. The engineers provided all the functions in accordance with our requirements. We also used all these functions.” They all stated that whenever they had any problem using the system, the problem was solved promptly.

### Impact of ICIS use on the nursing practice

The major benefits of the ICIS were improved quality and efficiency of nursing documentation, accuracy and efficiency of medication administration and overall nursing service quality and efficiency.

#### Improving quality of nursing documentation

Errors often occurred when nurses were required to hand write and draw diagrams to present the monitoring data. These could lead to inaccurate assessment of the condition of a critically ill patient. Using the ICIS, 100% accurate monitoring data were automatically generated; improving the accuracy of patient assessment.

Measured by the indicators of quality of nursing documentation including wrong data, missing data items, wrong calculations and legibility, the quality of electronic records significantly outperformed paper records. The rate of wrong data was reduced from 7.5% in the paper records to 1.6% in the electronic records. The rate of missing data dropped from 18.5% in the paper to 3.7% in the electronic records. The rate of wrong calculation decreased from 16.8% to 2.8%. The electronic records were completely legible, whereas 110 illegible data items were corrected in the paper records (see Table 2).

**Table 2:** A comparison of documentation errors in paper vs electronic records

Type of Records	Sample size	Data (n)		Data Item (n)		Calculation (n)		Legibility	
		Wrong (%)	Right	Missing (%)	Right	Wrong (%)	Right	Corrected	No Change
Paper	500	35 (7.5%)	465	78 (18.5%)	422	72 (16.8%)	428	110	390
Electronic	450	7 (1.6%)	443	16 (3.7%)	434	12 (2.8%)	434	0	450
Chi square		16.6		37.2		40.5		120.0	
*p* value		< 0.01		< 0.01		< 0.01		< 0.01	

Nurses were satisfied with the simplicity of electronic recording. Using the ICIS, the nurses could quickly and accurately complete nursing documentation about the treatment process for a patient. Only objective data and nursing process data were recorded, clearly and accurately. The extent of subjective assessment was believed to be reduced.

#### Reducing nursing documentation time and allowing more time on bed-side nursing tasks

Using paper, a nurse needed to handwrite monitoring data, which would take about 30 min per shift; whereas using the ICIS, the data were automatically recorded and generated into tables and diagrams for use. This completely freed nurses from these tedious, mechanical tasks, eliminating recording and calculation errors.

Using paper, retrieving data was time consuming. Using the ICIS, data could be quickly retrieved from the database. This led to the reduction of nursing pressure and the improvement of work efficiency (see Table 3). The nurses reported that they spent more time with patients, providing oral hygiene and skin care, teaching and comforting patients.

**Table 3:** A comparison of nursing documentation using paper vs electronic records

Item	Using Paper	Using ICIS
Recording the monitoring data	Hand writing, taking about 30 min per shift	Automatic generation of tables and diagrams
Reading patient’s information	Inconvenient to find paper records	Easy to retrieve data in ICIS
Accuracy of data captured from the monitoring devices	Missing data despite best effort	100% accurate
Storage of data	Paper records are easy to lose	Long-term storage in the database in the server
Data retrieval	Takes time and effort	Fast and directly retrieved from the database

According to the self-reported time usage data, using the paper records, a nurse would spend 3.80 h (*n* = 28, SD = 0.41) per 12 h shift to complete nursing documentation. Using the ICIS, the documentation time was significantly reduced to 2.05 h (*n* = 28, SD = 0.18, *t* = 25.19, *p* < 0.001).

When using the paper records, in a 24-h period, a nurse spent 20.20 h (*n* = 28, SD = 0.40) on bed-side nursing tasks. After using the ICIS, a nurse spent 21.95 h (*n* = 28, SD = 0.18) on bed-side nursing tasks (*t* = − 25.19, *p* < 0.001), an increase of 1.75 h in a 24-h period.

#### Improving accuracy and efficiency of medication administration

Using the ICIS, the whole process of medication administration was automated via barcode technology, including order transcription from the HIS system, verification and administration.

According to the clinical guideline, an order entry by a doctor first needed to be transcribed by a nurse to the nursing chart, then to be validated by the second nurse. Only after the two nurses both signed the medication chart to signal completion of the medication validation process could the order entry be sent to the pharmacy for dispensing.

In a doctor’s prescription, the dosage was recorded as the total amount of medication for a day. As a medicine was often needed to be administered several times for a patient in a day, it required a nurse to calculate the dosage for each medication, a source of calculation error.

As the ICIS directly populated the order entry data from the HIS, it eliminated the process of order transcription, an error prone process. The ICIS automatically presented the number of times that a medicine needed to be administered based on pattern analysis of medication administration for the common types of medicine. This provided an intuitive interface for a nurse to enter the dosage data. The nurse was also given the flexibility of changing administration time and dosage according to own discretion. Therefore, after implementation of the ICIS, the whole process of medication administration was automated and standardised. The error rate in transcription of medication prescription was believed to be reduced.

The ICIS also helped nurses to prioritize nursing treatment by the automatic classification of medications. For example, if a medication was placed in the temporary category, it signalled to a nurse that this medication had to be administered at the correct time. Long-term medication needed to be administered in order, at a fixed time interval, whereas administration of minor treatments, such as shoulder massage or oral hygiene care, could be done after the completion of oral medication and injection. Therefore, the nurses felt that they spent less time and less mental load on medication administration.

#### Impact on quality of nursing services

Data output from the ICIS was in various intuitive diagrams and charts, much more intuitive and convenient for a nurse to monitor and accurately understand the disease change process. The nurses reported that the saved time was used to observe the changes in a patient’s psycho-social status and to provide more personal care to the patients. The amount of oral hygiene, personal care and physiotherapy treatment to the patients was also increased after using the ICIS.

The study unit had always enjoyed high performance ratings in all mandatory nursing quality audit areas in the annual hospital nursing service evaluation (Table 4). The quantitative performance scores in all these areas improved after the introduction of the ICIS. The scores for ward management were improved from the baseline of 94.5 in 2012 to 99.7 in 2013, the year the ICIS was implemented. It increased to the highest level of 99.8 in 2014. The scores for routine nursing practice increased from 99 to 99.7, then 99.8 in the three years, respectively. The rate of improvement in disinfection and isolation was clear, from 94.1 in 2012 to 96.9 in 2013, then 99.8 in 2014, respectively. The score in nursing documentation was improved to 99.96 in 2014, starting from the baseline of 97.3. Infection rates dropped substantially, from 29.3 in 2012 to 20.0 in 2013, then 16.1 in 2013. Mortality rates dropped from 12.6 to 9.4, then 8.5 in the three years, respectively (see Table 4).

**Table 4:** A comparison of nursing performance before and after introduction of the ICIS

Item	2012 (Paper-records)	2013 (September, ICIS introduction)	2014 (Electronic records)
Ward management	94.5	99.7	99.8
Routine nursing practices	99	99.7	99.8
Disinfection and isolation	94.1	96.9	99.8
Nursing documentation	97.3	99.7	99.96
Infection rate	29.3	20.0	16.1
Mortality rate	12.6	9.4	8.5

## Discussion

Knowledge about the contemporary development of the ICIS worldwide, their usage and impacts on nursing services is beneficial for international advancement in building smart environments to transform ICU services in different healthcare context. This case study contributes new knowledge about how nurses contribute to the participatory design of an ICIS to support critical care nursing in a Chinese hospital. It describes the development and functionality of the ICIS, the nursing team’s use of the system, and the impact of system use on critical nursing care processes and outcomes. Both objective, performance evidence and nurses’ perceptions were gathered to provide a pre- and post-implementation comparison of quality and efficiency of nursing documentation, quality and efficiency of medication management, and impact on quality of nursing services.

Carayon et al. [31] find that factors related to technology design have significant effects on ICU nurses’ acceptance of electronic health records; however, there is little knowledge about how ICIS is designed and developed. This case study presents participative design, a useful approach for nurses to actively participate and contribute to the design and implementation of a useful ICIS for intensive care nursing services.

The success of the system, including its fit with nursing work processes and information needs was ensured by a committed, sustained collaboration in research and development of the ICIS by the ICU nurses, the hospital IT department and the software company developing the system. Once more, this case study proves that the active participation of the intended users in HIT design facilitates good design [22]. This effective, interactive system development and implementation process have also contributed to a lack of workforce resistance to IT introduction reported elsewhere [32]. The strong technical support provided by the on-site engineer, as well as strong peer support from the workmates, also met nurses’ learning needs.

### Improving the quality of nursing records, reducing time for documentation and increasing direct patient care time

Similar to findings by Bosman [13, 17] and Yusof [17], the use of the ICIS by the nurses in the study unit not only improved the quality of nursing records, but also reduced time spent on documentation and increased direct patient care time. All of the RNs who participated in the focus group discussions perceived that the nursing stress was reasonably relieved because the ICIS replaced nurses in meticulous, repetitive monitoring data entry and had also eliminated errors in this process. They were pleased to be able to spend more time to focus on providing direct nursing and personal care to the critically ill patients. Only two nurses left the ICU since the introduction of the ICIS in 2013 to 2016, suggesting that the nursing staff in the unit was generally satisfied with work in the unit, as observed by PY, who was external to the hospital. The nurses’ feedback was in agreement with the previous finding that the amount of time nurses spend with patients is linked to their job satisfaction, which is one of the critical factors for nurse retention [33].

### Improving the medication management process

Serious medication errors are common in hospitals and often occur during the process of order transcription and medication administration [34]. Heavy nursing workload and incomplete prescriptions are two significant risk factors for medication error [35]. Previous studies have found that use of bar-code electronic medical records can substantially reduce the rate of errors in order transcription and in medication administration [34, 36].

Standardisation of the order transcription and medication administration process was another mechanism that had led to the reduction of medication administration error in the ICU, as suggested by the previous study [35]. Although we did not have data about the change in medication error in this study, the reduction of annual patient mortality rate in the study unit in the year and one year after the ICIS introduction lend support to the improved quality and efficiency of order transcription and medication administration. This supports the wide adoption and usage of ICIS in medication management.

### Improving the quality of nursing care

The organization of nursing work in the study unit was improved with the automation of nursing documentation, particularly monitoring data collection, medication order transcription and management. The tasks that are markers of good nursing care, such as oral hygiene and skin care, teaching, and comforting patients [37], were increased.

### Limitation of the study

The limitation of the study was that only one ICU was studied to provide a bird’s eye view of the introduction of the ICIS in a Chinese hospital. A comparison of medication error rate before and after the implementation of the ICIS could provide useful, direct evidence about the effect of the ICIS on medication error, a key potential benefit of digitisation of ICU work. The improvement in nursing care quality cannot be fully attributed to the introduction of the ICIS. From its inception in 2009, only a few nursing staff left the ICU. Five-year daily hands-on nursing practice had led to a steady progress in team building, communication, improvement in individual nursing expertise, capacity and skill in intensive care nursing; an important contributor to the steady improvement in nursing performance.

Management of daily activities in ICU is challenging; ICU intensivists and nurses often need to make immediate ad hoc decisions to enable the optimal flow of activities [38]. Therefore, further research can be conducted to understand how intensivists and nurses actually work [39], and how to continuously build up the ICIS to support decision making of intensivists and nurses in ICU.

## Conclusions

Participatory design was a useful model for the nurses, the hospital IT department and the software company to collaboratively research and develop a user-centred, intelligent ICU environment. Use of the ICIS had brought benefits including improving quality and efficiency of nursing documentation, accuracy and efficiency of medication administration, overall improvement in nursing service quality, and improvement in working conditions and quality of work life for the nurses. Further research can be focused on understanding how to enhance the functionality of the ICIS to support decision making of intensivists and nurses, and nursing performance measurement in ICU.

## Abbreviations

APACHE II score: Acute care physiology and chronic disease evaluation
DON: Director of Nursing
EMR: Electronic medical records
HIS: Hospital information system
ICIS: Intensive care information system
ICT: Information and communication technology
ICU: Intensive care unit
LIS: Laboratory information system
MODS score: Comprehensive assessment of multiple organ dysfunction
PACS: Picture archiving and communication system
RN: Registered nurse
SOFA score: Sequential organ exhaustion evaluation system
